# Supplementary material for: miR-302 regulates pancreatic progenitor pool and pancreatic size
Source: Biol Open. 2026 Jan 2;15(1):bio062353. doi: 10.1242/bio.062353 (PMC12805655; doi:10.1242/bio.062353)
Supplement: Supplementary information [file biolopen-15-062353-s1.pdf]

Table S1.

Available for download at
https://journals.biologists.com/bio/article-lookup/doi/10.1242/bio.062353#supplementary-data

Table S2.

Available for download at
https://journals.biologists.com/bio/article-lookup/doi/10.1242/bio.062353#supplementary-data

Table S3.

Available for download at
https://journals.biologists.com/bio/article-lookup/doi/10.1242/bio.062353#supplementary-data

Table S4.

Available for download at
https://journals.biologists.com/bio/article-lookup/doi/10.1242/bio.062353#supplementary-data

Table S5.

Available for download at
https://journals.biologists.com/bio/article-lookup/doi/10.1242/bio.062353#supplementary-data

Table S6.

Available for download at
https://journals.biologists.com/bio/article-lookup/doi/10.1242/bio.062353#supplementary-data

Table S7.

Available for download at
https://journals.biologists.com/bio/article-lookup/doi/10.1242/bio.062353#supplementary-data

Table S8.

Available for download at
https://journals.biologists.com/bio/article-lookup/doi/10.1242/bio.062353#supplementary-data

Biology Open • Supplementary information
